# Anthropometric Parameters in Asthmatic Children and the Relationship of Childhood Asthma with Height, Weight and Body Mass Index

**DOI:** 10.9734/jpri/2019/v27i530181

**Published:** 2019-05-22

**Authors:** Nasrin Bazargan, Shokouh Hamidifar, Ali Khalouei, Gholamreza Sedighi

**Affiliations:** 1 Department of Pediatrics, Afzalipour Medical Center, School of Medicine, Kerman University of Medical Sciences, Kerman, Iran.; 2 Department of Community Medicine, School of Medicine, Kerman University of Medical Sciences, Kerman, Iran.

**Keywords:** Children, asthma, anthropometric index, a corticosteroid

## Abstract

**Background:**

Asthma as a chronic disease may affect the growth process. The aim of this study was to investigate the anthropometric indices in 2–18 years old children with asthma and compare them with the control group.

**Patients and Methods:**

In a case-control study, 150 asthmatic children with age of 2–18 years as case group and 300 age- and sex-matched healthy children as control group were randomly included. The height, weight, and body mass index (BMI) of both group measured by the standard method and Z score was calculated. Data were analyzed using SPSS, chi-square and analysis of variance.

**Results:**

Totally, 290 boys (64.4%) and 160 girls (35.6%) with mean age of 6.58±2.82 years were evaluated. Case group had significantly lower height compared to the healthy control group (117.00±0.17 cm vs. 121.00±0.15 cm respectively, P=0.025). No significant differences were detected in weight (23.13±9.75 kg vs. 24.62±10.36 kg, P=0.145) and BMI (16.32±3.10 kg/m^2^ vs. 16.28±3.16 kg/m^2^, P=0.900) between case and control groups, respectively. There were no significant relationships between normal and abnormal Z scores of height, weight and BMI in case and control group (P>0.05).

**Conclusion:**

Despite 4 cm difference between the age of two groups, no differences in height, weight ad BMI between two groups may be due to good control of the disease in the case group or lack of significant growth related effect of asthma.

## 1. INTRODUCTION

Growth is a complex process that is influenced by genetic factors, hormones, nutritional status, physical activity and chronic diseases [1]. Growth disorder is one of the most common and important health problems in childhood. Asthma is a chronic inflammatory disease of the airways affected more than 300 million people worldwide [2] and caused about 250,000 death at 2011 [3]. Childhood asthma is an important cause of emergency attendance, hospitalization and school absorbability and is considered as one of the growth problems [4].

Asthma as a chronic respiratory disease disrupts oxygenation as well as medications used to treat asthma including corticosteroids, either orally or as an inhaler is affected growth. More than 70 years ago, it has been found that allergic children presented a growth arrest and their height and bone maturity were affected [5]. In the early 1970s, inhaled corticosteroids were introduced for the treatment of asthma and now are the best effective medications for this inflammatory disease in both adults and children [6]. However, their side effects on the growth of children are topics of concern yet. Due to the high medical burden of childhood asthma and the lack of evidence about the growth-related effects of this disease in our province, Kerman, one of the biggest province in Southern of Iran, the aim of this study was to compare the anthropometric indices in asthmatic children and the control non-asthmatic group.

## 2. MATERIALS AND METHODS

### 2.1 Patients

In this case-control study, 150 asthmatic children visited at the Asthma and Allergy Clinic of Afzalipour Medical Center from October 2016 to March 2017 were randomly selected as the case group. The ratio of control to case group was considered as 2:1. Therefore 300 healthy children were included as a control group by a multi-stage sampling from different schools and kindergartens in Kerman city. Informed consent was taken from the participants or their parents. Children with any growth-interfering diseases/disorders such as diabetes mellitus, hypothyroidism, convulsion and consumption of anti-convulsant or other drugs which impaired growth were excluded.

### 2.2 Evaluations

All participants were visited by asthma and allergy specialist. Asthma status was evaluated in the case and control groups using history, clinical examination and spirometry. Weight and height of each participant were measured using a digital scale and stadiometer, respectively. BMI was calculated as weight/(height)^2^ and expressed as kg/m^2^. A questionnaire contained demographic data including age, sex, educational status, and parent occupation and income were filled. Growth indices were analyzed based on national Z score as:

- Z score of height for age: normal (−2< and <+3) and abnormal (<−2 and >+3)
- Z score of weight for age: normal (−2< and <+1) and abnormal (<−2 and >+1)
- Z score of BMI for age: normal (−2< and <+1) and abnormal (<−2 and >+1)

### 2.3 Statistical Analysis

All data were statistically analyzed using SPSS version 22. Frequency (percentage) for qualitative data and mean and standard deviation (SD) for quantitative data were reported. Two independent t-test and one way ANOVA were used to analyze differences. P<0.05 was considered a significant difference.

## 3. RESULTS

The age range of 2–18 years was enrolled in the study. Total mean ± SD (range) of age, height, weight, and BMI of our participants were 6.58 ± 2.82 years (2.00 – 17.33 years), 119.00 ± 16.25 cm (84.00 – 170.00 cm), 24.12 ± 10.18 kg (2.90 – 63.50 kg), and 16.29 ± 3.13 kg/m^2^ (9.70 – 31.40 kg/m^2^), respectively. Demographic characteristics of our case and control groups are presented in Table 1. Most of our participants were male (64.4%) and first birthday rating (54%) and had ≤ 4 family members (74.6%) employed father (71.8%), unemployed mother (67.7%) and academic educated father (49.4%).

Most of our asthmatic patients had > 2500 g birth weight (85.3%), > 10,000,000 IRR income (52%), no asthma attack (74.0%), no hospitalization (86.0%), no familial history of asthma (47.3%), used corticosteroids (78.0%) and had mild persistent asthma (72.7%) (Table 2).

**Fig. 1.**
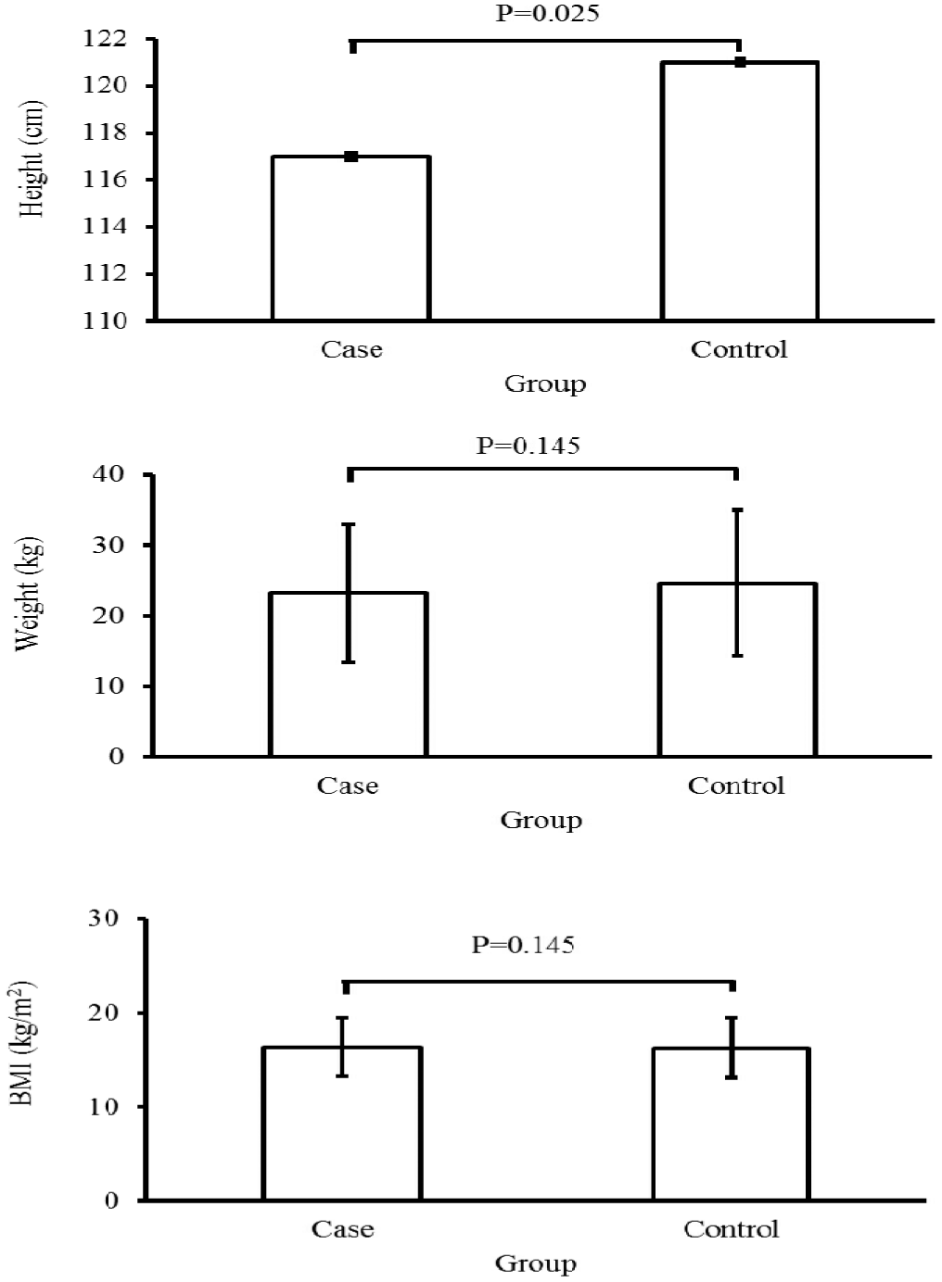
Between-group comparison of height, weight, and BMI

Our asthmatic patients had significantly lower height compared to the control group (P=0.025). No significant differences were detected in weight and BMI between two groups (P>0.05, Fig. 1). Also, no significant differences were seen in Z scores of height, weight, and BMI based on age between control and case group (P>0.05, Table 3).

Comparison of height, weight, and BMI in asthmatic patients based on different demographic and disease-related variables is presented in Table 4. Just significant relationship was detected between having hospitalization and lower weight (P=0.02).

As revealed from ANOVA values, no significant associations were seen between frequency and percentage of normal and abnormal Z scores of height, weight, and BMI based on age in asthmatic patients and some disease-related variables (P > 0.05, Table 5).

## 4. DISCUSSION

In the present study, we evaluated and compared height, weight and BMI of asthmatic and control children. We found that asthmatic patients had a significantly lower height (about 4 cm) compared to the control group. However, Z scores of height, weight, and BMI as indices of changes over time showed no significant differences between asthmatic and healthy children. Also, having hospitalization in asthmatic patients had a significant relationship with a lower weight. However, we found no associations between weight, BMI and their Z scores with asthma.

In a randomized clinical trial study, Mohammadi et al. investigated the effects of treatment with inhaled corticosteroids on the growth of 70 asthmatic children of 6–12 years old and compared them with 70 healthy children as a control group. They found no significant negative effects for the use of beclomethasone and fluticasone on linear growth of asthmatic children [4]. This is in opposition to our findings of mean height but in line with no change in Z score of height in our study. There is a clear controversy between the results of different studies. In an ancient study at1969, Dawson and colleagues by evaluation of 121 asthmatic children among 2743 school children with ages of 10–15 years found that their weight and height tended towards being below average for gender and age [7]. On the other hand, it has been reported that bone mineral density in children with asthma was not influenced by long-term treatment with inhaled fluticasone propionate as a corticosteroid [8]. Some studies describe that asthma itself and administration of corticosteroids for its treatment affect a child’s growth. For instance, some studies reported impaired baseline growth in children with severe or intractable asthma which is due to the disease itself [9–12]. However, another study showed normal baseline growth in asthmatic children [13–17]. It seems that the severity of asthma also is important in growth impairment. Evaluation of 7411 primary schoolchildren who had parent-reported respiratory symptoms confirmed a negative relationship between the height and asthma severity in the last 1 years [18]. Another study with 173,034 participants includes 8531 asthmatics found a 0.7 cm mean reduction in the height at 18 years of age in asthmatic patients, compared to those without asthma plus negative correlation of severity of asthma with the height [19]. Finally, a study of 92,143 17-year-old participates including 3410 asthmatic patients found a slight decrease in weight, height and BMI in asthmatics plus inverse association between all three growth variables and asthma severity, especially in boys [20]. The growth impairment effects of asthma itself can be described as delayed puberty, severity and control of the disease, and impaired endocrine function [21].

About adverse effects of asthma treatment by corticosteroids, in a randomized clinical trial of 360 children with mild persistent asthma (119 beclomethasone, 120 montelukast, and 121 placeo) in USA, montelukast did not affect linear growth, whereas the growth rate with beclomethasone was significantly decreased during 1 year of treatment [22]. In another randomized trial in Denmark, 42 children with age of 6–11 years with mild persistent asthma (37 montelukast and 34 budesonide), montelukast did not significantly affect short-term lower leg growth rate in prepubertal children [23]. Three recent systematic reviews also confirmed the growth adverse effects of corticosteroids therapy for asthma. In a Cochrane review of 25 trials, it has been confirmed that compared to placebo or non-steroidal drugs, inhaled corticosteroids (ICS) significantly decreased both linear growth velocity and mean height during a one-year treatment period [24]. Another Cochrane review [25] and a systematic review and meta-analysis [26] also confirmed such effects. The possible mechanisms are inhibition of growth hormone secretion, insulin-like growth factor-1 (IGF-1) bioactivity, collagen synthesis, and adrenal androgen production, down-expression of growth hormone receptors, and having a direct growth-retarding effect on the growth plates [6,24,27].

## 5. CONCLUSION

Although previous reports suggested that asthma itself and ICS as treatment adversely impaired the child’s growth, we found no such effects in our participants may be due to good control of the disease. It seems more studies with high sample size in different geographical regions are needed in this controversial topic.

## Figures and Tables

**Table 1.** Total and group-specific demographic characteristics of our patients

Variables		Case group	Control group	Total
Sex	Girl	52 (34.7)	108 (36.0)	160 (35.6)
	Boy	98 (65.3)	192 (64.0)	290 (64.4)
Birthday rating	First	88 (85.7)	155 (51.7)	243 (54.0)
	2^nd^ and more	62 (41.3)	145 (48.3)	207 (46.0)
Family members	≤ 4	114 (76.0)	252 (76.0)	336 (74.6)
	≥ 5	36 (24.0)	78 (24.0)	114 (25.4)
Father’s job	Employed	49 (32.7)	274 (91.3)	323 (71.8)
	Unemployed	101 (67.3)	26 (8.7)	127 (28.2)
Mother’s job	Employed	19 (12.7)	127 (42.3)	146 (32.4)
	Unemployed	131 (87.3)	173 (57.7)	304 (67.7)
Father’s education	Intermediate and lower	52 (34.7)	62 (20.7)	114 (25.4)
	High school and diploma	44 (29.3)	69 (23.1)	113 (25.2)
	Academic	54 (36.0)	168 (56.2)	222 (49.4)

**Table 2.** Some demographic characteristics of asthma patients

Variables		Frequency (percentage)
Birth weight	< 2500 g	22 (14.7)
	> 2500 g	128 (85.3)
Income	< 10,000,000 IRR	72 (48.0)
	> 10,000,000 IRR	78 (52.0)
Asthma attack	Yes	39 (26.0)
	No	111 (74.0)
Hospitalization	Yes	21 (14.0)
	No	129 (86.0)
Familial history	No	71 (47.3)
	In first degree relatives	29 (19.3)
	In other relatives	50 (33.3)
Use of corticosteroids	Yes	117 (78.0)
	No	33 (22.0)
Disease severity	Mild intermittent	41 (27.3)
	Mild persistent	108 (72.7)

**Table 3.** Comparison of Z scores of height, weight, and BMI based on age in control and case group

Z scores		Case group	Control group	P value
Height for age	Normal	139 (92.7)	291 (97.0)	0.34
	Abnormal	11 (7.3)	9 (3.0)	
Weight for age	Normal	139 (92.7)	293 (97.7)	0.13
	Abnormal	11 (7.3)	7 (2.3)	
BMI for age	Normal	123 (82.0)	252 (84.0)	0.34
	Abnormal	27 (18.0)	48 (16.0)	

**Table 4.** Comparison of height, weight, and BMI in asthmatic patients based on different demographic and disease-related variables

Variables		Height	P	Weight	P	BMI	P
Father’s education	Intermediate and lower	1.20±0.19	0.17	24.53±9..97	0.39	16.42±3.30	0.71
	High school and diploma	1.14±0.13		21.88±8.76		16.00±3.02	
	Academic	1.15±0.16		22.80±10.31		16.40±2.99	
Mother’s education	Intermediate and lower	1.19±0.23	0.40	25.13±12.11	0.35	16.54±2.62	0.89
	High school and diploma	1.17±0.14		23.03±9.91		16.25±3.81	
	Academic	1.14±0.14		22.07±7.91		16.26±2.51	
Father’s job	Employed	1.17±0.16	0.94	24.47±11.62	0.24	16.93±3.61	0.89
	Unemployed	1.16±0.17		22.48±8.70		16.01±2.79	
Mother’s job	Employed	1.18±0.16	0.71	24.04±8.61	0.66	16.59±2.05	0.68
	Unemployed	1.16±0.17		23.00±9.93		16.28±3.22	
Birthday rating	First	1.17±0.15	0.88	22.97±9.47	0.80	16.30±3.39	0.92
	2^nd^ and more	1.16±0.18		23.36±10.22		16.34±2.65	
Birth weight	< 2500 g	1.19±0.22	0.44	23.67±11.40	0.77	15.70±2.64	0.31
	> 2500 g	1.16±0.15		23.04±9.49		16.42±3.17	
Income	< 10,000,000 IRR	1.19±0.16	0.14	23.21±8.66	0.92	16.10±3.00	0.41
	> 10,000,000 IRR	1.14±0.16		23.06±10.70		16.52±3.19	
Asthma attack	Yes	1.13±0.14	0.13	20.94±7.23	0.10	15.72±2.42	0.16
	No	1.18±0.17		23.90±10.42		16.52±3.29	
Hospitalization	Yes	1.07±0.15	0.08	18.59±6.83	0.02	15.49±2.11	0.18
	No	1.18±0.16		23.87±9.98		16.45±3.22	
Use of corticosteroid	Yes	1.17±0.16	0.25	23.15±9.21	0.96	16.20±3.01	0.40
	No	1.13±0.18		23.07±11.65		16.71±3.41	
Disease	Mild intermittent	1.13±0.17	0.13	22.48±10.28	0.18	16.47±3.01	0.89
severity	Mild persistent	1.17±0.16		23.22±9.49		16.27±3.15	

**Table 5.** Frequency (percentage) of normal and abnormal Z scores of height, weight, and BMI based on age in asthmatic patients according to some disease-related variables

Variables						Z scores				
			Height			Weight			BMI	
		Normal	Abnormal	P	Normal	Abnormal	P	Normal	Abnormal	P
Asthma attack	Yes	36 (92.3)	3(7.7)	0.17	36 (92.3)	3(7.7)	0.19	33 (84.6)	6 (15.4)	0.11
	No	103 (92.8)	8 (7.2)		103 (92.8)	8 (7.2)		90 (81.8)	21 (18.9)	
Hospitalization	Yes	19 (90.5)	2 (9.5)	0.13	18 (85.7)	3 (14.3)	0.14	19 (90.5)	2 (9.5)	0.19
	No	120 (93.0)	9(7.0)		121 (93.8)	8 (6.2)		104 (80.6)	25 (19.4)	
Familial history	Yes	73 (92.4)	6 (7.6)	0.57	73 (92.4)	6 (7.6)	0.60	66 (83.5)	66 (83.5)	0.40
	No	66 (93.0)	5 (7.0)		66 (93.0)	5(7.0)		57 (80.3)	57 (80.3)	
Use of corticosteroid	Yes	112 (95.7)	5 (4.3)	0.73	110 (94.0)	7(6.0)	0.56	96 (82.1)	21 (17.9)	0.54
	No	27 (81.8)	6 (18.2)		29 (87.0)	4 (12.1)		27 (81.8)	6 (18.2)	
Disease severity	Mild intermittent	37 (90.2)	4 (9.8)	0.41	39 (95.1)	2 (4.9)	0.14	32 (78.0)	9 (22)	0.11
	Mild persistent	101 (93.5)	7(6.5)		100 (92.6)	8 (7.4)		91 (84.3)	17 (15.7)	
